# RNA sequencing-based screen for reactivation of silenced alleles of autosomal genes

**DOI:** 10.1093/g3journal/jkab428

**Published:** 2021-12-21

**Authors:** Saumya Gupta, Denis L Lafontaine, Sebastien Vigneau, Asia Mendelevich, Svetlana Vinogradova, Kyomi J Igarashi, Andrew Bortvin, Clara F Alves-Pereira, Anwesha Nag, Alexander A Gimelbrant

**Affiliations:** Department of Cancer Biology and Center of Cancer Systems Biology, Dana-Farber Cancer Institute, Boston, MA 02215-5450, USA; Department of Genetics, Harvard Medical School, Boston, MA 02115, USA; Broad Institute of MIT and Harvard, Cambridge, MA 02142, USA; Department of Cancer Biology and Center of Cancer Systems Biology, Dana-Farber Cancer Institute, Boston, MA 02215-5450, USA; Department of Cancer Biology and Center of Cancer Systems Biology, Dana-Farber Cancer Institute, Boston, MA 02215-5450, USA; Department of Genetics, Harvard Medical School, Boston, MA 02115, USA; Department of Cancer Biology and Center of Cancer Systems Biology, Dana-Farber Cancer Institute, Boston, MA 02215-5450, USA; Skolkovo Institute of Science and Technology, Moscow 121205, Russia; Department of Cancer Biology and Center of Cancer Systems Biology, Dana-Farber Cancer Institute, Boston, MA 02215-5450, USA; Department of Cancer Biology and Center of Cancer Systems Biology, Dana-Farber Cancer Institute, Boston, MA 02215-5450, USA; Department of Cancer Biology and Center of Cancer Systems Biology, Dana-Farber Cancer Institute, Boston, MA 02215-5450, USA; Department of Cancer Biology and Center of Cancer Systems Biology, Dana-Farber Cancer Institute, Boston, MA 02215-5450, USA; Department of Genetics, Harvard Medical School, Boston, MA 02115, USA; Broad Institute of MIT and Harvard, Cambridge, MA 02142, USA; Department of Cancer Biology and Center of Cancer Systems Biology, Dana-Farber Cancer Institute, Boston, MA 02215-5450, USA; Department of Cancer Biology and Center of Cancer Systems Biology, Dana-Farber Cancer Institute, Boston, MA 02215-5450, USA; Department of Genetics, Harvard Medical School, Boston, MA 02115, USA; Broad Institute of MIT and Harvard, Cambridge, MA 02142, USA; Altius Institute for Biomedical Sciences, 2211 Elliott Ave, Seattle, WA 98121, USA

**Keywords:** gene regulation, DNA methylation, allelic expression

## Abstract

In mammalian cells, maternal and paternal alleles usually have similar transcriptional activity. Epigenetic mechanisms such as X-chromosome inactivation (XCI) and imprinting were historically viewed as rare exceptions to this rule. Discovery of autosomal monoallelic autosomal expression (MAE) a decade ago revealed an additional allele-specific mode regulating thousands of mammalian genes. Despite MAE prevalence, its mechanistic basis remains unknown. Using an RNA sequencing-based screen for reactivation of silenced alleles, we identified DNA methylation as key mechanism of MAE mitotic maintenance. In contrast with the all-or-nothing allelic choice in XCI, allele-specific expression in MAE loci is tunable, with exact allelic imbalance dependent on the extent of DNA methylation. In a subset of MAE genes, allelic imbalance was insensitive to DNA demethylation, implicating additional mechanisms in MAE maintenance in these loci. Our findings identify a key mechanism of MAE maintenance and provide basis for understanding the biological role of MAE.

## Introduction

In mammalian cells, the maternal and paternal gene copies tend to make an equal contribution to transcription (Mohammadi *et al.* 2017). However, several allele-specific modes of gene regulation provide important exceptions. One such mode is genomic imprinting, where the allelic choice is determined by the parent of origin in about 200 mammalian genes (Tucci *et al.* 2019). Another is X-chromosome inactivation (XCI), which randomly silences one of the two copies of the X chromosome in females (Galupa and Heard 2018), affecting over 800 X-linked genes. Additionally, olfactory sensory neurons express one allele of 1 out of ∼1000 olfactory receptor genes (Chess *et al.* 1994).

**Figure 1:**
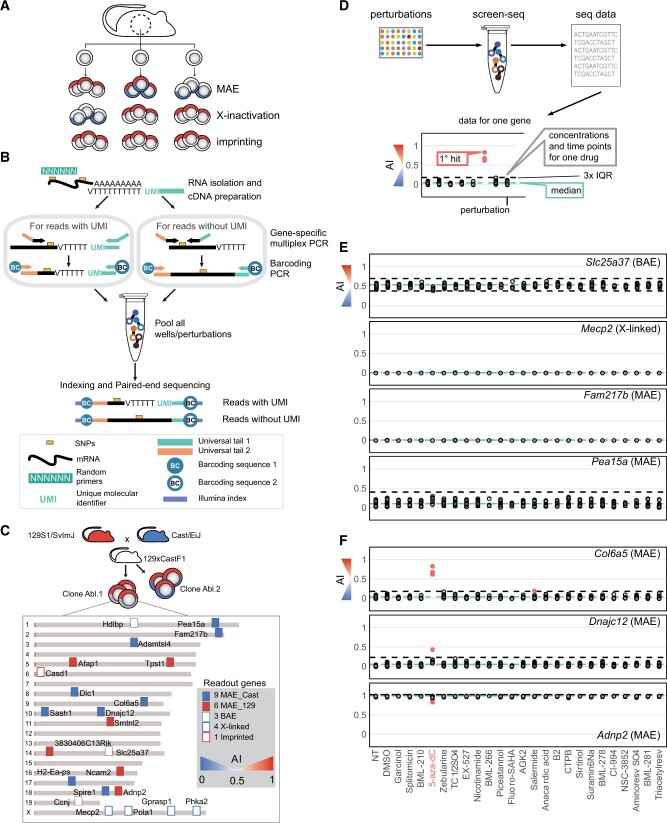
Perturbations that reactivate silenced alleles of genes with monoallelic expression identified using screening-by-sequencing. (A) Different epigenetic modes of monoallelic expression. Note that while imprinting is uniform across cells, X chromosome inactivation and autosomal MAE result in epigenetic clonal mosaicism. (B) Outline of Screen-seq methodology. Top to bottom: Cells are lysed in-plate, and in each well, RNA is isolated using SPRI beads. Two types of SNPs between parental genomes for the readout genes are targeted: those close to the poly-A tail enabling use of the UMI (*left*) and the rest that were targeted with two gene-specific primers with universal tails (*right*). Well-encoding is performed using primers targeting common adapters coupled with barcodes (BC1 and BC2). Then, all wells are pooled, Illumina sequencing adapters are added, and the pooled library is sequenced. (C) Twenty-three genes assayed in Screen-seq and their distribution in the mouse genome. AI of target genes in Abl.1 clone is reflected by the marker color. Centromeres (*brown*) on the left. (D–F) Screen setup and results for a representative set of readout genes. Each of the tested 43 drugs were used in three final concentrations: 1, 10, and 20 µM in 1% DMSO. Fresh media and drugs were replaced every 2 days; 19 of the 43 drugs where no live cells were evident after 6 days were not further considered. Cells were collected for the remaining 24 drugs on days 7, 14, and 21 and processed for Screen-seq. (D) Presentation of primary screen results. All time and concentration points for a single drug shown in the same column. Each point shows allelic imbalance in one condition [AI=(129 counts)/(129 + Cast counts)]. *Blue dashed line*: mean AI for a gene across all conditions; *black dashed lines*: (Q1 – 3×IQR) and (Q1 + 3×IQR) (inter-quartile range); *red* points: outlier AI values (1˙ hits/primary hits). (E) Genes showing no AI change in any condition. (F) genes with significant changes in some conditions. Screen-seq results for all readout genes are in Supplementary Figure S3 and corresponding data are in Supplementary Table S5.

Our view of allele-specific gene regulation was greatly expanded by the discovery of widespread monoallelic autosomal expression (MAE) (Gimelbrant *et al.* 2007). Like XCI, MAE involves a random choice of the active allele during development, resulting in an epigenetic mosaic (Chess 2016). Also like XCI, the allelic choice in MAE genes is mitotically stable; however, MAE genes can be expressed from both alleles in a subset of clonal lineages (Figure 1A). MAE had been observed in clonal populations of every cell type assessed (Gimelbrant *et al.* 2007; Jeffries *et al.* 2012; Li *et al.* 2012; Zwemer *et al.* 2012; Nag *et al.* 2013; Deng *et al.* 2014; Eckersley-Maslin *et al.* 2014; Gendrel *et al.* 2014) and most MAE genes are highly cell-type specific (Nag *et al.* 2015a). Cumulatively across cell types, an estimated 4000 human genes are subject to MAE (Savova *et al.* 2016b), including genes implicated in cancer and neurodevelopmental disorders.

Multiple lines of evidence indicate that MAE has a significant impact on organismal function. MAE has been shown to result in dramatic functional differences between otherwise similar cells; for example, the function of B cells in mice heterozygous for *Tlr4* depends on which allele is active in a given cell (Pereira *et al.* 2003). Evolutionary and population-genetic analyses indicate conservation of the MAE status between human and mouse (Zwemer *et al.* 2012; Nag *et* al. 2015b), and selective advantage for individuals heterozygous for MAE genes (Savova *et al.* 2016a, 2017). The prevalence of cell surface molecules among proteins encoded by MAE genes prompted the hypothesis that MAE leads to increased variation in responses to extrinsic signals between otherwise similar cells (Savova *et al.* 2016a).

Lack of knowledge about the underlying mechanism of MAE has severely limited research on MAE function. At present, no perturbation is known to affect the maintenance of allele-specific silencing in any MAE locus (da Rocha and Gendrel 2019). MAE status correlates with histone modifications and DNA methylation in the gene body and putative regulatory regions (Nag *et al.* 2013; Eckersley-Maslin *et al.* 2014; Gendrel *et al.* 2014; Nag *et* al. 2015b; Xu *et al.* 2017). However, inhibiting the DNA methyltransferases was reported to not affect the allelic imbalance (AI) in any of the tested MAE genes (Eckersley-Maslin *et al.* 2014; Gendrel *et al.* 2014), arguing against a mechanistic role of DNA methylation in MAE (da Rocha and Gendrel 2019).

To understand the mechanistic basis of MAE, we devised a novel strategy for screening by targeted RNA sequencing and performed a small molecule screen for perturbations that affect AI in expression in any of the 23 targeted genes across the mouse genome. We found that inhibition of *Dnmt1* methyltransferase-dependent DNA methylation reactivated silenced alleles in many MAE loci, showing that DNA methylation plays a major role in the MAE mitotic maintenance. At the same time, other MAE loci showed no significant changes in allele-specific expression upon DNA demethylation, suggesting that MAE is mechanistically heterogeneous. We conclude that DNA methylation acts as a tunable mechanism for AI in MAE loci, controlling allele-specific transcription quantitatively, as opposed to an on–off switch.

## Methods

### Cell culture

v-Abl pro-B clonal cell lines Abl.1 derived previously from 129S1/SvImJ × Cast/EiJ F1 female mice (Zwemer *et al.* 2012) were cultured in Roswell Park Memorial Institute medium (Gibco), containing 15% FBS (Sigma), 1× L-Glutamine (Gibco), 1× Penicillin/Streptomycin (Gibco), and 0.1% β-mercaptoethanol (Sigma).

### Drug treatment

The SCREEN-WELL^®^ Epigenetics arrayed drug library was used for initial drug screening and was purchased from Enzo Life sciences (BML-2836) and included 10 mM solutions of the compounds in DMSO. Cells from the Abelson clone Abl.1 were seeded in 96-well plates (10^5^ cells/well); the component drugs (Supplementary Table S3) were diluted in growth medium at final concentrations of 1, 10, and 20 µM to encompass a wide range of potentially pharmacologically active concentrations. Cultures were treated for 21 days; every second day, media was aspirated and replaced with fresh media with appropriate drug. Cells were collected on days 7, 14, and 21. Prior to collection, cells were visually inspected under phase contrast microscope, and wells with no visible live cells were discarded. Of the 43 drugs, 19 led to loss of viability on the first day of collection and were not further considered.

For following up hits from the initial drug screen, validation experiments were performed. For this, 5-aza-2'-deoxycytidine (5-aza-dC, Sigma, A3656) was diluted in DMSO at a concentration of 10 mM and Abl.1 cells were plated in 200 µl of medium supplemented with concentration range of 10 nM to 20 µM 5-aza-dC. Cells were treated for a total of 7 days where media was changed every 2 days and samples of ∼1 × 10^5^ cells were harvested for RNA extractions on days 2, 5, and 7. Viable cells were counted using trypan blue solution (Gibco™) on Countess II FL Automated Cell Counter machine (Life Technologies) and noted in Supplementary Figure S5. For all treatments, drugs were solubilized in DMSO and dilutions were made to ensure the final DMSO added to cultures was 1% (v/v).

For exposure/recovery experiment (Figure 3), 5-aza-dC was dissolved in water; 1.5 × 10^6^ cells were seeded in medium supplemented with 5-aza-dC concentrations of 0.2 μM, 0.5 μM, and 1 μM for 2 days. Control cells were not treated with any drug and only grown in growth medium. After 2 days, cells exposed to the drug were washed and were grown only in growth medium (without drug, called recovery phase) for the rest of the days. We observed that Abelson lymphoid cell lines show ideal growth at densities of 1–2 × 10^6^ cells/ml. During recovery, we observed overcrowding in low concentrations of 5-aza-dC and in control well and so cells were passaged on days 5, 7, and 9 to maintain optimal densities. Percentages of viable cells were calculated and noted in Supplementary Table S6.

### RNA and DNA preparation

For all clonal Abelson lymphoid cell lines, RNA was extracted from cells using a magnetic bead-based protocol using Sera-Mag SpeedBeads™ (GE Healthcare). Isolated RNA was DNase-treated with RQ1 DNase (Promega). First strand cDNA synthesis was done using Episcript™ RNase H-reverse transcriptase (Epicentre) where RNA samples were primed with random hexamers (NEB). Both DNase treatment and cDNA synthesis were performed using manufacturer specifications with minimal modifications. For RNA preparation from mouse spleen, cells were extracted by crushing the whole spleen using the back of 1 ml syringe plunger in 40 µM nylon filter and washing the strainer with 1× phosphate-buffered saline (Sigma) to collect cells. Cells from spleen were spun down and RNA was extracted using Trizol reagent (Invitrogen). Genomic DNA extractions for testing the sensitivity of Screen-seq were performed using the salting out method (Miller *et al.* 1988). Real-time quantitative PCRs were performed using iTaq™ Universal SYBR^®^ Green Supermix (BioRad) using manufacturer’s protocol on a 7900HT Fast Real-Time PCR system (Applied Biosystems Inc.). All primers used in this study were ordered from Integrated DNA Technologies and their sequences are listed in Supplementary Table S2.

### Screen-seq methodology

A targeted sequencing method, similar to that described in Nag *et al.* (2013), was used to assay multiple genes simultaneously for assessing allele-specific expression. Here, we assayed 23 genes. The assay involved RNA extraction, cDNA synthesis (Figure 1B), two rounds of PCR amplification, and Illumina sequencing. After magnetic bead-based RNA purification, cDNA synthesis was performed within each well of a 96-well plate, separately using EpiScript™ Reverse Transcriptase (EpiCentre Biotechnologies) using both random hexamers (NEB) and Unique Molecular Identifier (UMI)-tagged oligo-dT primer with universal tail (Supplementary Table S2) using manufacturer’s instructions. Half the portion cDNA products were transferred to a separate 96-well plate. Gene-specific multiplex PCR are performed in both the plates using Phusion U multiplex Master Mix (ThermoFisher, F562L, Waltham, MA). Two types of multiplexed readouts were generated within each plate: readouts without UMI and readouts with 3′-UMI. For the multiplex readouts without UMI, target genes that contain the single nucleotide polymorphisms (SNPs) differentiating the maternal and paternal allele were amplified using gene-specific primer pairs containing one of two universal tails (UT1 or UT2, Supplementary Table S2). For the multiplex readouts with 3′-UMI, the forward primers were gene-specific and contained universal tail UT2 (Supplementary Table S2). They were always positioned near the SNP of interest. Reverse primer for these genes were complimentary to the universal tail UT1. These readouts were always constrained to the 3′ end of the transcript. These two types of multiplex readouts were not generated for all readout genes. A list of the readout genes for which the multiplex assay was used is given in Supplementary Table S1. *MPprimer* v1 (Shen *et al.* 2010) was used to design the non-UMI multiplex PCR assay. We computationally generated an input form that would (1) constrain our SNP(s) of interest within 135 base pairs from one end of the amplicon, (2) mask repetitive regions, (3) prevent the design of primers pairs that exist within more than one exon, and (4) ensure that the total fragment size for each readout falls within 250–500 base pairs. Once the gene-specific primer sequences were designed, the universal tails were added. Primers generated were tested for specificity and primer dimerization using *MFEprimer v2.0* (Qu *et al.* 2009) and also experimentally validated. The two groups of multiplex products from the gene-specific PCR were combined and carried over as templates to the second PCR, which was performed using Phusion High-Fidelity DNA Polymerase (New England Biolabs Inc., M0530L, Ipswich, MA) while barcoding each well/perturbation separately. These reactions use primers that target the universal tails (UT1 and UT2) of the readouts amplified in the first multiplex PCR and add a six-nucleotide barcode, a seven-nucleotide spacer, and an Illumina primer dock (Supplementary Table S2). Combinatorial barcoding was achieved by using a pair of unique forward and reverse primers, which tag each sample with a unique barcode combination. These barcode combinations allowed pooling of samples in the subsequent steps of the assay. Once pooled, the readout library was cleaned up using magnetic beads at a bead to sample ratio of 1:2 to get rid of primer dimer bands < 150 bp in size. The sample was then carried over as a template into a third PCR reaction which adds Illumina adapters.

We observed high accuracy of multiplexing and barcoding steps of Screen-seq by comparing the AI calculated from Screen-seq and expected AI for a range of pure 129 and Cast parental genomic DNA mixes for all genes (Supplementary Figure S1). A good correlation was observed between the reads with UMI and without UMI for readout genes tested using both methods. For this, Screen-seq was performed for a range of RNA mixes from pure 129 and Cast mice spleen. AI calculated from Screen-seq for *Adamtsl4* and *Adnp2* showed good correlation with the expected AI, and also between UMI and non-UMI assays (Supplementary Figure S2). *Smtnl2* and *Dnajc12* showed low expression in mice spleen tissue and hence comparison could not be made. Finally, the assays for genes we selected had to combine successfully in multiplexed PCR.

### Screen-seq data analysis

After Screen-seq libraries were prepared as described above, they were sequenced at the UMass Boston and Center for Cancer Systems Biology sequencing core on Illumina HiSeq 2500 and MiSeq, respectively, using four-color reagent kits. From the P7 adapter end, 65 nt were sequenced (Read 1), including one of the two barcodes for encoding plate wells (and the UMI, where appropriate). From the P5 adapter, the remaining 135 nt were sequenced (Read 2), covering the second well-encoding barcode and the cDNA amplicon containing the interrogated SNP. In addition, standard Illumina barcodes were used to distinguish individual plates within the overall pooled library, with demultiplexing before further processing. Reads were aligned using *bowtie2 v.2.2.9* (Langmead and Salzberg 2012) against mm10 mouse genome assembly. The resulting BAM files were processed using custom Perl scripts (available at github.com/gimelbrantlab/drug-screen-seq) to extract allele-specific, UMI-corrected counts for each gene and each well.

To identify primary hits (outliers in Figure 1, E and F), the allele-specific counts were analyzed using custom R scripts. Briefly, for each gene, point AI estimates for all drug conditions were considered together to determine median AI and the interquartile range (IQR = Q3–Q1, with Q1 and Q3 the 25th and 75th percentiles, respectively). Observations with counts under 30 were filtered out (an observation consists of allelic counts for one gene in one well). A common practice for identification of outliers is to use values below Q1 – 1.5×IQR or above Q3 + 1.5×IQR (Tukey 1977). We used a more stringent threshold of 3×IQR to reduce the likelihood of false positive hits. Complete results can be found in Supplementary Table S5.

### Droplet digital PCR

Droplet digital PCRs (ddPCRs) were performed on QX200 ddPCR system (BioRad) for absolute quantification of 129 and Cast alleles using manufacturer-recommended settings. C1000 Touch™ thermal cycler was used to perform amplification within droplets. SNP-specific TaqMan assays (IDT; sequences in Supplementary Table S2) were designed manually. We first validated all TaqMan assays experimentally using homozygous Cast and 129 cDNA and optimized reaction conditions for each assay using Abl.1 clonal cell line cDNA, including Tm of each primer–probe mix by performing thermal gradient PCR. Finally, we tested the specificity of this method by using known quantities of left kidney cDNA from homozygous 129 and Cast mice parents and comparing it with the estimated AI from ddPCR. To determine the false-positives, we made twofold dilutions of these samples starting from 1 ng cDNA till its 1/16th dilution. Results demonstrated our ability to precisely measure AI in samples with 30 copies/µl using ddPCR. cDNA was prepared from around 100,000 cells and 8 μl template cDNA (one-fourth of eluted sample) was used per reaction. Gating for clusters with maternal and paternal alleles was decided by comparing the fluorescence intensity individually for the maternal and paternal probes in homozygous 129 and Cast tissue samples. Data were processed using QuantaSoft v.1.6 (Bio-Rad). Inverse fractional abundance given displayed by the QuantaSoft software was divided by 100 and noted as AI measurement [mat/(mat + pat)] from ddPCR.

### shRNA infection

Two shRNA vectors targeting *Dnmt1* (SHR000038801.1_TRC001.1 and SHR000373188.1_TRC005.1) and a control empty vector (NUL003.3_TRC021.1) packaged in lentiviral vectors obtained from the Genetic Perturbation Platform at the Broad Institute were tested. The optimal multiplicity of infection (MOI) was determined by infecting Abl.1 cells with pLKO_TRC060 lentiviral vector expressing eGFP. Abl.1 cells were infected with three shRNA vectors (two targeting Dnmt1 and one control) individually on day 1 at the optimal MOI under normal growth conditions in the presence of 8 µg/ml polybrene and spun at 800×g for 90 min at 37°C. The next day, the media was changed and media containing 2 µg/ml of puromycin was added on day 2. Selection was maintained continuously afterwards, and media changes were done every 2–3 days. Cells were harvested on days 12 and 19 after infection, and RNA was extracted.

## Results

### Screening-by-sequencing approach for changes in allele-specific gene expression

To screen for reactivation of a silenced allele, we looked for shifts in AI (the fraction of one allele over the total) upon drug exposure. In order to increase the likelihood of detecting AI shifts among genes with potentially different regulation, our screening approach would ideally combine the ability to assess multiple readout genes, sensitivity to AI changes, and the throughput to process multiple samples after exposure to an array of perturbations.

We designed a screening-by-sequencing strategy, Screen-seq, to satisfy these requirements. In cells with heterozygous genomes, allele-specific expression can be assessed without the need for any engineered reporters and by relying on the detection of SNPs. Precision and sensitivity of the AI measurement in RNA sequencing critically depend on the depth of SNP coverage (Mendelevich *et al.*^2021^). Sequencing of SNP-containing amplicons from multiplexed RT-PCR as the readout allows for very deep coverage and thus a highly precise AI measurement.

The experimental flow of Screen-seq is outlined in Figure 1B. Cells were grown and lysed in 96-well plates; RNA was isolated using magnetic beads, and cDNA was synthesized with a mix of random primers and oligo-dT primers with UMIs (Kivioja *et al.* 2011; Islam *et al.* 2014). This mix allowed targeting of two types of SNPs in the next step, multiplex PCR: SNPs close to the 3′-end enable the use of oligo-dT-UMIs followed with a gene-specific primer, while other SNPs were targeted with two gene-specific primers in random-primed cDNA. Next, plate- and well-encoding barcodes were added using PCR. The reactions from all the wells were pooled, Illumina adaptors added, and the pooled library was sequenced. Finally, SNP counts were assigned to specific genes, and barcodes to specific plates and wells with a specific perturbation.

To allow analysis of MAE genes, which show different AI in different clones, we performed our screen in a monoclonal line of pro-B cells (Abl.1). We have previously characterized allele-specific expression in several such lymphoid clones, including Abl.1 and other clones used in this study (Zwemer *et al.* 2012; Nag *et* al. 2015a). These cells were derived from female mice, immortalized using the Abelson murine leukemia virus (Rosenberg *et al.* 1975), and cloned through single-cell sorting. Cells were from 129S1/SvImJ × Cast/EiJ F1 mouse cross, with the median distance between SNPs in the non-repetitive genome of ∼80 bp and thus almost 80% of genes containing at least one informative SNP.

For readout, we selected 27 SNPs in 23 target genes across the genome, including 15 clone-specific MAE genes as well as three biallelic, one imprinted, and four X-inactivated loci (Figure 1C, Supplementary Table S1). The selected MAE genes showed AI > 0.9 or AI < 0.1 in the Abl.1 clone [AI=(129 counts)/(129 + Cast counts)], while showing opposite bias or biallelic expression in another clone, Abl.2 (Zwemer *et al.* 2012; Nag *et al.* 2015a). Targeted MAE genes spanned a range of expression levels and extent of allelic bias in the screening clone, Abl.1; some showed complete silencing of one allele (such as *Afap1*, AI = 1), while others showed strong but incomplete bias (such as *Dlc1*, AI = 0.1).

We first tested that these assays were able to detect changes in AI. Since no perturbations are known that can change AI in any locus, much less in all targeted loci, for the control experiments, we titrated known mixes of genomic DNA from liver tissue of the parental mouse strains, 129S1/SvImJ and Cast/EiJ. Expected and measured AI were highly concordant (Supplementary Figure S1). We also compared AI sensitivity for UMI and non-UMI assays, by designing both types of assays for a subset of genes where the position of SNPs allowed that. For this, we used mixes prepared from total RNA from the spleens of the mice of the parental mouse strains. AI measurements were highly concordant between the UMI and non-UMI assays (Supplementary Figure S2).

Based on these pilot experiments, we concluded that Screen-seq can be used for sensitive detection of AI changes in the targeted loci.

### Identification of perturbations that affect allele-specific gene expression

Clone-specific MAE has been associated with specific chromatin signatures, *i.e*., combinations of histone modifications in human and mouse cells (Nag *et al.* 2013; Eckersley-Maslin *et al.* 2014; Nag *et* al. 2015a), suggesting that chromatin modifying mechanisms might be involved in MAE maintenance. We thus assessed the impact on AI in the targeted loci of treatment with a set of 43 small molecules with known effects on the activity of the enzymes involved in the deposition and removal of methylation and acetylation marks on histones and DNA (Supplementary Table S3). Abl.1 cells in 96-well plates were exposed for 21 days to individual drugs in regular growth conditions. Each drug was applied in three final concentrations (1, 10, and 20 µM in 1% DMSO). Controls were untreated cells and cells with only solvent (1% DMSO) added. Fresh media (with or without drugs, as appropriate) was replaced every 2 days. On days 7, 14, and 21, aliquots of cells were removed for analysis.

For 19 of the 43 drugs, no live cells were evident after 6 days, at any drug concentration (see Methods). Each cell collection thus involved only 72 wells with treated cells (24 remaining drugs at three concentrations, drug names in Supplementary Table S3 and Figure 1F*X*-axis) and 24 wells with controls (12 untreated and 12 vehicle-treated cells). Taken together, in this Screen-seq experiment, we assessed 7776 experimental points (allele-specific measurements of 27 SNPs × 96 wells × 3 time points). With a targeted RNA-seq library, only a very moderate amount of sequencing was needed to reach the coverage depth required for sensitive allele-specific analysis. At 1000 reads per experimental point, fewer than 10 × 10^6^ sequenced fragments were needed for the entire screen.

As potential primary hits, we identified conditions resulting in outlier AI values (Figure 1, D–F; see Methods for details). AI measurements were highly uniform for some genes (*e.g*., *Fam217b* or *Mecp2*) across drug concentrations and time points, while there was more variation in other genes (*e.g*., *Pea15a* or *Col6a5*). To allow for variation in assay sensitivity, each readout gene was analyzed independently of the rest. Outliers were identified using highly stringent criteria (see Methods).

As expected for stably maintained allele-specific expression, in the untreated cells, there were no outliers for any of the readout genes. The most pronounced outliers (red in Figure 1F) were observed for three MAE readout genes in the presence of drug 5-aza-dC. There were also significant AI shifts in single readout genes after exposure to histone deacetylase modulators Salermide and BML-278 (Supplementary Table S4, complete Screen-seq results are in Supplementary Figure S3 and Table S5). The magnitude of the observed shifts varied between genes and conditions, including drug concentration and exposure times. Among the targeted loci, the most striking example was a shift in *Col6a5* gene from baseline paternal bias of AI ≈ 0.1 in the control to maternal bias of AI ≈ 0.8 after 7 days in the presence of 1 µM 5-aza-dC (Figure 1F). The magnitude of this shift was surprising, since the after-treatment AI was not close to 50:50 balance; instead, the AI changes from one extreme to close to another extreme. Significant, but more subtle, shifts were observed after exposure to the same drug in the MAE genes *Adnp2* (from AI = 1 to AI = 0.8) and *Dnajc12* (AI ≈ 0.1 to AI ≈ 0.2). In other tested genes, no AI shift was observed in 5-aza-dC (Supplementary Figure S3).

### 5-aza-dC affects allele-specific expression of autosomal MAE genes via DNA demethylation

**Figure 2:**
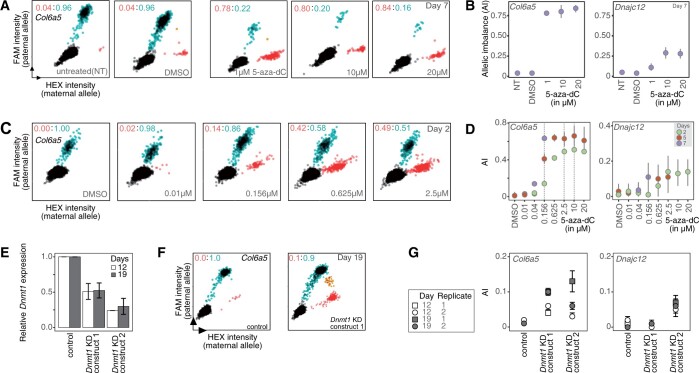
Hit validation for 5-aza-dC. (A and B) Confirmation of Screen-seq results for 5-aza-dC-treated cells using an orthogonal method to measure AI. cDNA samples from day 7 of screening were assessed using ddPCR with allele-specific fluorescent probes. (A) Scatterplots for 20,000 droplets targeting the readout gene, *Col6a5*. 5-aza-dC concentration is shown in the plots. *Black*: empty droplets; *blue*: droplets with the Cast paternal allele amplified (labeled by FAM fluorophore); *red*: droplets with the 129 maternal allele amplified (labeled by HEX fluorophore). Ratio of red: blue droplets are shown. AI value written in *red* is the maternal AI. Note that the double-positive droplets (orange) contained both maternal and paternal templates; a small number of such double-positives is expected with higher concentrations of biallelic template. These droplets are ignored in the quantitative analysis. (B) *left—*summary of AI measurements shown in (A) for *Col6a5* on day 7; *right—*summary of AI measurements for *Dnajc12* on day 7. (C and D) Biological replicate of Abl.1 cells were treated with 5-aza-dC and AI was measured using ddPCR. (C) Scatterplots representation as shown in (A) after 2 days of exposure. (D) Summary of AI measurement for *Col6a5* (left) and *Dnajc12* (right) after 2, 5, and 7 days of exposure (denoted by color). Gray vertical dashed lines for *Col6a5* dose-response were used to determine “low,” “medium,” and “high” 5-aza-dC concentrations for the future experiments. Results for readout gene *Adnp2* are in Supplementary Figure S4. (E–G) Analysis of *Dnmt1* knock-down (KD) in Abl.1 cells. (E) Real-time quantitative PCR (RT-qPCR) analysis of *Dnmt1* relative expression (expression in the empty vector control, normalized to *Nono*, taken as 1.0). Abl.1 cells were transduced with an empty plKO vector (control) or with two separate *Dnmt1* shRNA knockdown constructs (*Dnmt1* KD construct 1 or 2) and grown for 2 days. Transduced cells were then selected by growing in the presence of a selection antibiotic for an additional 17 days. RT-qPCR quantification was performed on cells collected 19 days after transduction. Mean and SEM for three technical replicates are shown. (F) Representative scatterplots show AI measurement for *Col6a5* in the transduced Abl.1 cells. AI was measured using ddPCR. (G) Summary of the AI measurement for *Col6a5* (left) and *Dnajc12* (right) after *Dnmt1* KD.

We focused on characterizing the strongest primary hit, 5-aza-dC, a classic DNA demethylation agent (Jones and Taylor 1980). To validate it, we performed several sets of experiments. First, we took advantage of the fact that the Screen-seq protocol leaves enough RNA and cDNA for additional analyses. We measured AI in the same samples using an orthogonal method, ddPCR (a highly sensitive approach to measuring allelic frequencies, Kamitaki *et al.* 2018). In addition to using a different readout method, we assessed different SNPs than those used for Screen-seq for the same genes (Supplementary Table S2). Using cDNA from cells treated with 1, 10, and 20 µM of 5-aza-dC for 7 days, we performed ddPCR to assess reactivation of the silenced maternal allele of the *Col6a5* and *Dnajc12* genes. Confirming the results from Screen-seq, ddPCR measurements showed a similarly striking shift in *Col6a5* AI from a paternal bias to maternal bias (AI = 0.1–0.8) after 7 days in 1 µM 5-aza-dC (Figure 2, A and B). Also confirming the Screen-seq results, AI for *Dnajc12* gene showed relaxation toward a more biallelic expression, with AI shifting from 0 to 0.1 in 1 µM 5-aza-dC and to 0.3 in 20 µM 5-aza-dC in 7 days (Figure 2B and Supplementary Figure S4).

In biological replicate experiments, the Abl.1 clonal cells were exposed to a range of concentrations of 5-aza-dC for varying times. Using ddPCR, we observed that the maternal allele of *Col6a5* was reactivated in a dose- and time-dependent manner (Figure 2, C and D). AI shifts for *Dnajc12* and *Adnp2* were also concordant with those observed in Screen-seq (Figure 2D, Supplementary Figures S4 and S5). Taken together, these observations show that 5-aza-dC causes a shift in AI in a subset of MAE genes.

A closely related compound, 5-aza-cytidine (5-aza-C), is also a well-known demethylating agent, although less potent and toxic than 5-aza-dC (Christman 2002). Since 5-aza-C was not one of the perturbagens tested in our screening, we assessed whether it had a similar effect as 5-aza-dC on AI changes. Within 2 days of treatment with 10 µM 5-aza-C, the AI of *Col6a5* shifted from 0 to 0.2, and to 0.6 after 5 days in 2 µM 5-aza-C (Supplementary Figure S6). Another MAE readout gene, *Dnajc12*, showed a shift in AI from 0 to 0.1 within 2 days in 2 µM 5-aza-C. This further supports the role of DNA methylation in MAE maintenance.

5-aza compounds at high concentrations are cytotoxic and cause cell cycle arrest (Zhu *et al.* 2004). We asked whether shifts in AI in the target genes might be due to nonspecific cytotoxicity. In the presence of 2% DMSO, higher than the 1% concentration used as a drug solvent, the Abl.1 clonal cells viability was reduced to 34% after 2 days, similar to their viability after 5 days in 2.5 µM 5-aza-dC (Supplementary Figure S5). In contrast to the AI shifts in the presence of 5-aza-dC and 5-aza-C (Figure 2D and Supplementary Figure S6), no changes in AI were observed for the MAE readout genes, *Col6a5* and *Dnajc12*, in 2% DMSO (Supplementary Figure S7), indicating that AI shifts are not a generalized feature of cells under stress.

To test if the effect of 5-aza-dC on allele-specific expression was specific to inhibition of methyltransferase activity, we assessed changes in AI in response to the knock-down of *Dnmt1*, the main maintenance methyltransferase in mammals (Smith and Meissner 2013). Abl.1 cells transduced with *Dnmt1* shRNA constructs showed twofold and fourfold decrease in *Dnmt1* RNA abundance (Figure 2E), and the corresponding partial reactivation of silenced alleles of *Col6a5* and *Dnajc12* (Figure 2, F and G). This result is consistent with the role of Dnmt1 in AI maintenance.

Taken together, these observations strongly suggest that *Dnmt1*-dependent DNA methylation is a molecular mechanism involved in AI maintenance of MAE genes.

### Changes in AI are long-term and tunable

**Figure 3:**
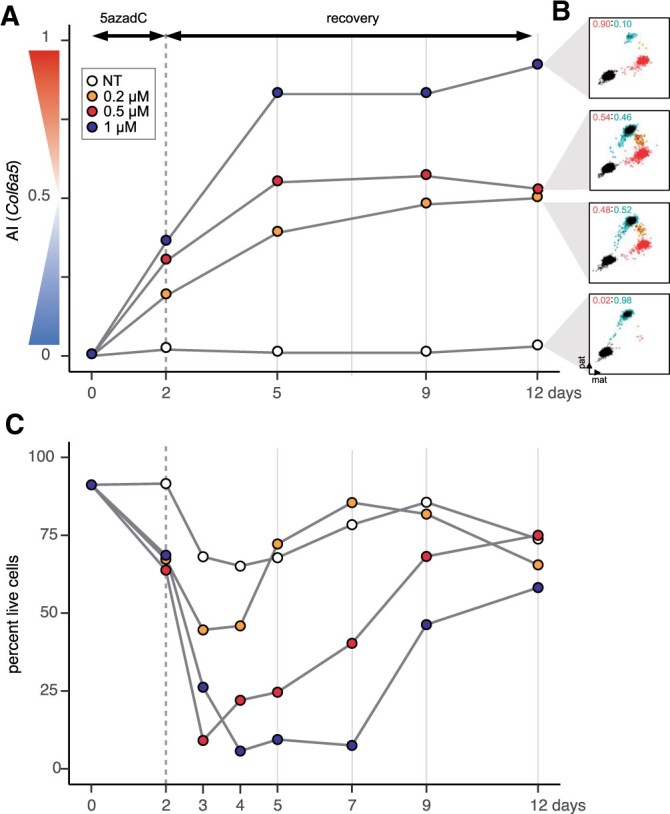
Long-term changes in the mitotic memory of allelic imbalance after exposure to 5-aza-dC and recovery. (A) 5-aza-dC exposure/recovery experiment in Abl.1 cells. Cells were exposed to 0.2, 0.5, or 1 µM 5-aza-dC in growth medium for 2 days (gray dashed vertical line). Control cells were not treated (NT) with drug and only grown in growth medium. After 2 days, cells exposed to the drug were washed and grown only in growth medium (without drug, called recovery phase) for the rest of the days. Cells were collected on days 2, 5, 7, 9, and 12 (gray solid vertical line). Days are shown on the *X*-axis. AI measurements for *Col6a5* across time points were made using ddPCR and are shown on the *Y*-axis. (B) ddPCR scatterplots for *Col6a5* on day 12 after recovery [as summarized in (A)]. See Supplementary Figure S8 for ddPCR scatterplots for readout gene *Dnajc12*. (C) Cells recover and grow after drug removal. Percent of live cells (by automated Trypan blue assay) was determined for the cells shown in (A) on the indicated days.

We asked if the changes in AI were mitotically stable and capable of long-term maintenance, the hallmark of autosomal MAE. To address this question, we performed a treatment-and-recovery experiment (Figure 3 and Supplementary Figure S8). First, Abl.1 cells (with doubling time of ∼12 h) were exposed to 5-aza-dC in growth medium; after 2 days, cells were washed and incubated further in the regular growth medium (recovery period).

After 2 days of treatment and 3 days of recovery, AI of readout genes reached levels that remained stable through days 9 and 12. Figure 3, A and B shows the AI readout for *Col6a5* gene (similar results were seen with *Dnajc12* gene, Supplementary Figure S8). Importantly, cells recovered and began dividing (Figure 3C), showing that the observed AI values were a new mitotically stable state rather than a readout from arrested cells.

This shows that AI shifts resulting from 5-aza-dC treatment were maintained over multiple subsequent cell divisions. Such stability is consistent with DNA methylation as the molecular mechanism that maintains the long-term memory of AI state of MAE genes in clonal cells. A continuing AI shift over the first 3 days of recovery is consistent with the cell population right after treatment being heterogeneous and containing some remaining fraction of cells with the readout gene in the initial state of AI ≈ 0. By day 5, that fraction would be replaced by cells in the new stable state of DNA methylation, and the new state would then be maintained through days 9 and 12.

We observed in other experiments (see Figure 2) that the extent of allelic shift was dose dependent. Notably, the eventual stable AI states after recovery were also dependent on the 5-aza-dC concentration during cell exposure (Figure 3). This shows that 5-aza-dC-dependent allele-specific regulation acts not as an on–off switch, but rather in a tunable manner, with multiple stable intermediate states.

## Discussion

Using a screening-by-sequencing approach, we identified DNA methylation as a key mechanism involved in the mitotic maintenance of monoallelic expression in clonal lymphoid cell lineages of mammalian cells. Dnmt1-dependent maintenance DNA methylation offers a straightforward explanation for MAE stability, since it is a very stable form of molecular memory: as an extreme example, cytosine methylation in the *Cryptococcus* genome has apparently been maintained for millions of years in the absence of *de novo* methylation (Catania *et al.* 2020).

Not all assessed MAE genes were affected by DNA demethylation, suggesting that MAE maintenance for some loci involves other mechanisms in addition to (or instead of) DNA methylation. This offers one likely explanation of the previous observations that DNA demethylating agents did not affect AI in any of the several assessed MAE genes (Eckersley-Maslin and Spector 2014; Gendrel *et al.* 2014). Consistent with the idea of additional mechanisms of MAE maintenance, a SIRT1 activator, BML-278, and a sirtuin inhibitor, salermide, appeared as other primary hits in our screen (see Supplementary Figure S3), suggesting that expanded application of the Screen-seq strategy can uncover such additional mechanisms. Additionally, a recent report suggested a role for CTCF-mediated chromatin dynamics in regulating allelic transcriptional states (Chandradoss *et al.* 2020).

We asked whether responsiveness of transcription AI to DNA demethylation clearly correlated with the presence of CpG islands near the promoter region of genes. For genes with a strong response, *Col6a5* and *Dnajc12*, closest CpG islands were ∼200 and ∼50 kb (Supplementary Figure S10). Conversely, non-responding gene Fam217b had a CpG island overlapping its transcription start site (Supplementary Figure S10). This is consistent with the notion that the relevant changes in DNA methylation occurred in the regions other than CpG islands. Identification of these specific regions whose methylation affects allele-specific expression of MAE genes is a subject for an in-depth study.

Limitations of this study include the following. The scalability of the screening strategy we described is limited by the depth of multiplexing of the targeted PCRs. The screen is potentially prone to false negatives. Negative result in the screen, *i.e*., no clear change of AI in a gene, could be due to nonspecific factors such as cell toxicity at high drug concentrations, masking biological response. We did perform multiple validations to confirm our primary hit to overcome this limitation; however, more experiments will be required for other Screen-seq hits. Relatedly, the specific conditions (concentrations and exposure times) used in the screen cover only a small fraction of their feasible permutations. The *Dnmt1* knockdown experiments (see Figure 2G) are consistent with the role of DNMT1 in the AI maintenance, but a definitive proof requires further experiments.

Significant impact of DNA demethylation drugs on allele-specific expression in lymphocytes is of particular importance since both 5-aza-2′-dC and 5-aza-C are used in the clinic to treat acute leukemia and other malignancies (Christman 2002). Notably, concentrations of these compounds in our experiments (0.2–1.0 µM; Figure 3) are similar to that measured in the patients’ plasma (5-aza-dC at ∼60 ng/ml, about 0.25 µM, Karahoca and Momparler 2013). Our findings thus imply that DNMT inhibitors likely affect gene regulation in patients in ways that would be undetectable without allele-specific analysis. This suggests that quantitative analyses of allele-specific gene regulation in polyclonal and monoclonal cell populations should lead to new clinically relevant insights.

## Data availability

The authors affirm that all data necessary for confirming the conclusions of the article are present within the article, figures, and supplementary material. Main Supplementary File contains Supplementary Figures S1–S10. Supplementary Tables S1–S6 are given separately as excel files. Source code used to analyze Screen-seq data is available on github at https://github.com/gimelbrantlab/drug-screen-seq.

Supplementary material is available at *G3* online.

## Supplementary Material

jkab428_Table_S1

jkab428_Table_S2

jkab428_Table_S3

jkab428_Table_S4

jkab428_Table_S5

jkab428_Table_S6

jkab428_Supplemental_Material
