# Supplementary material for: RNA sequencing-based screen for reactivation of silenced alleles of autosomal genes
Source: G3 (Bethesda). 2021 Dec 21;12(2):jkab428. doi: 10.1093/g3journal/jkab428 (PMC9210281; doi:10.1093/g3journal/jkab428)
Supplement: jkab428_Supplemental_Material [file jkab428_supplemental_material.pdf]

MAIN SUPPLEMENTARY FILE

Contents of this file
All Supplementary Figures

SUPPLEMENTARY FIGURES

**Fig.S1**	Concordance between expected vs. measured allelic imbalance (AI) from Screen-seq.	2
**Fig.S2**	Concordance between UMI vs non-UMI based AI measurement from Screen-seq.	3
**Fig.S3**	Screen-seq results for 23 readout genes.	4
**Fig.S4**	Relaxation towards biallelic expression in Dnajc12 allele imbalance in Abl.1 cells on 5-aza-dC treatment.	6
**Fig.S5**	Dose response curve for Adnp2 and cell viability of Abl.1 clone in 'biological replicate experiment'.	7
**Fig.S6**	5-aza-cytidine (5-aza-C), a closely related compound to 5-aza-dC, causes shifts in allelic imbalance of MAE readout genes Col6a5 and Dnajc12.	8
**Fig.S7**	Inducing cell toxicity in clone Abl.1 by growth in high concentration of DMSO (2%) does not change allelic imbalance of Col6a5 and Dnajc12.	9
**Fig.S8**	5-aza-dC exposure-and-recovery experiment in Abl.1 cells – readout gene Dnajc12.	10
**Fig.S9**	Change in Xist allele imbalance in Abl.1 cells is very subtle on exposure to 5-aza-dC treatment.	11
**Fig.S10**	Changes of allele-specific expression of autosomal genes appear independent of CpG islands.	12

SUPPLEMENTARY TABLES

**Table.S1**	Description of readout genes used in Screen-seq, AI in monoclonal lymphoid cell lines Abl.1 and Abl.2.	Provided separately
**Table.S2**	Primer information. For Screen-seq, ddPCR and RT-qPCR.	Provided separately
**Table.S3**	Drug library tested on Abl.1 clonal lymphoid cell line.	Provided separately
**Table.S4**	Readout genes: All drug screen hits.	Provided separately
**Table.S5**	Complete Screen-seq results.	Provided separately
**Table.S6**	Cell viability and total cell numbers for the 'exposure and recovery experiment'.	Provided separately

Supplementary Figure S1

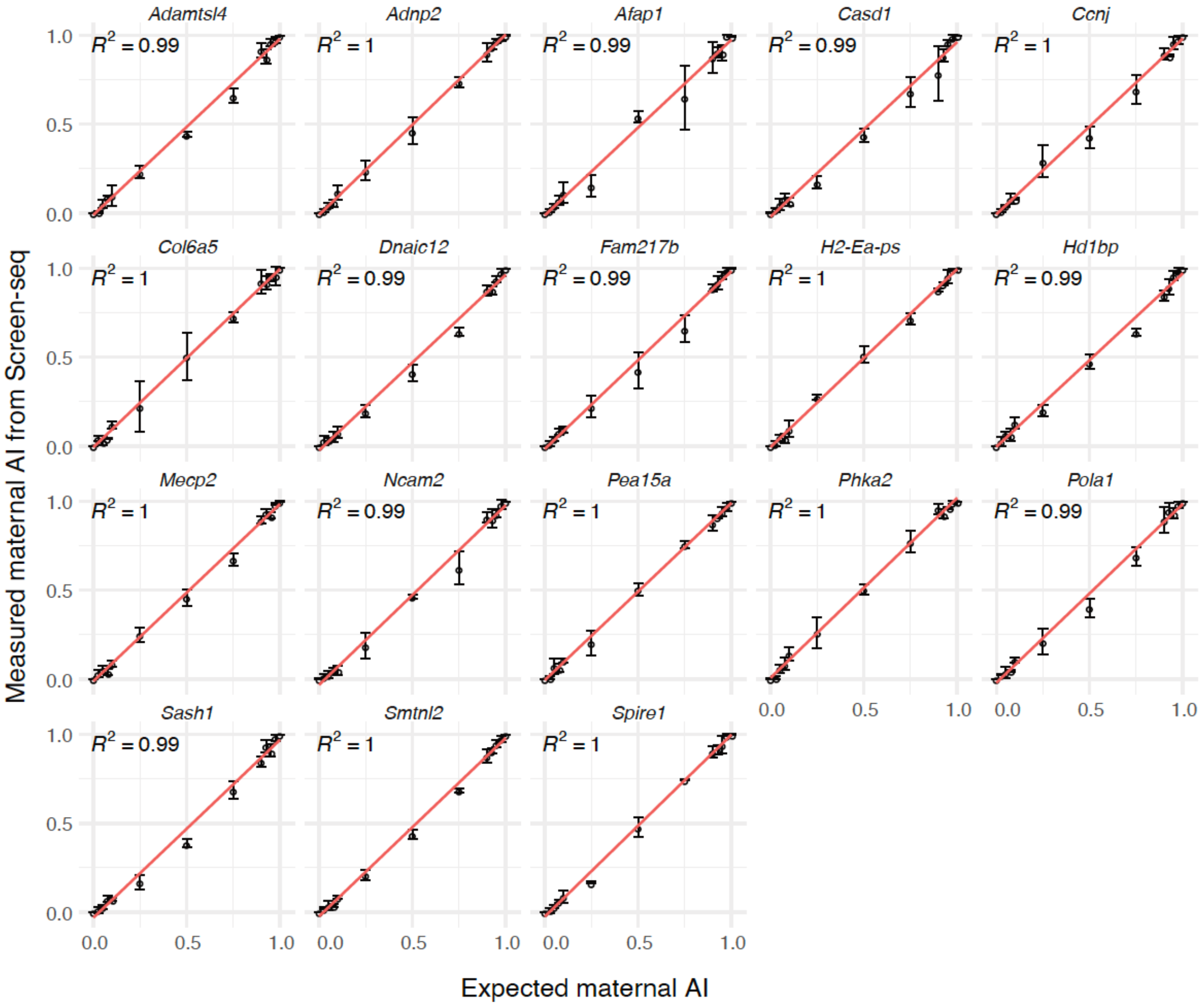

Fig.S1. Concordance between expected *vs.* measured allelic imbalance (AI) from Screen-seq. Since no perturbations are known that can change AI in any locus, much less in all targeted loci, for the control experiments we titrated known mixes of genomic DNA from liver tissue of the parental mouse strains, 129S1/SvImJ and Cast/EiJ. Allelic imbalance was measured as AI= [129 counts]/[129 + Cast counts]. Genomic DNA (gDNA) mixes ranging from 0 to 0.1 and 0.9 to 1 in 0.025 increments, as well as 0.25, 0.5, and 0.75, were made from parental liver tissue and shown on X-axis (Expected maternal AI). For AI measured from Screen-seq (Measured AI, Y-axis), data points are an average of 3 biological replicates and error bars show standard deviation. *Red* line denotes linear fit to smoothen all data points. Expected and measured AI were highly concordant ($R^2 \geq 0.99$) at >1000 reads/SNP for readout genes.

Supplementary Figure S2

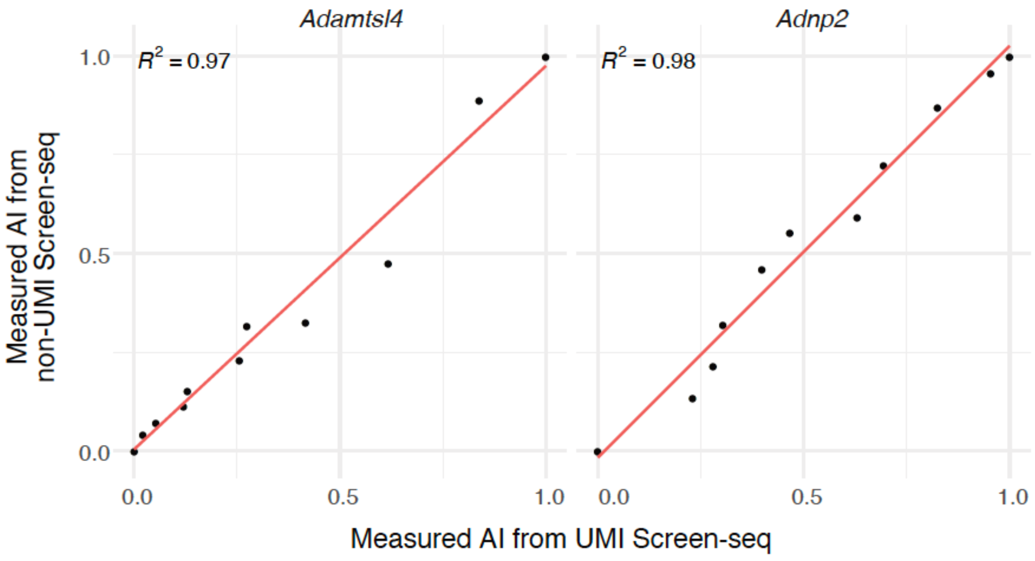

Fig.S2. Concordance between UMI vs non-UMI based AI measurement from Screen-seq.
We compared AI sensitivity for UMI and non-UMI assays, by designing both types of assays for a subset of genes where the position of SNPs allowed that. For this, we used mixes prepared from total RNA from the spleens of the mice of the parental mouse strains. Both types of assays were designed for a subset of genes (*Adamtsl4, Adnp2, Dnajc12, Smtnl2*) where the position of SNPs allowed that. AI measurements were highly concordant between the UMI and non-UMI assays ($R^2 \geq 0.97$) at >1000 reads/SNP.

Supplementary Figure S3

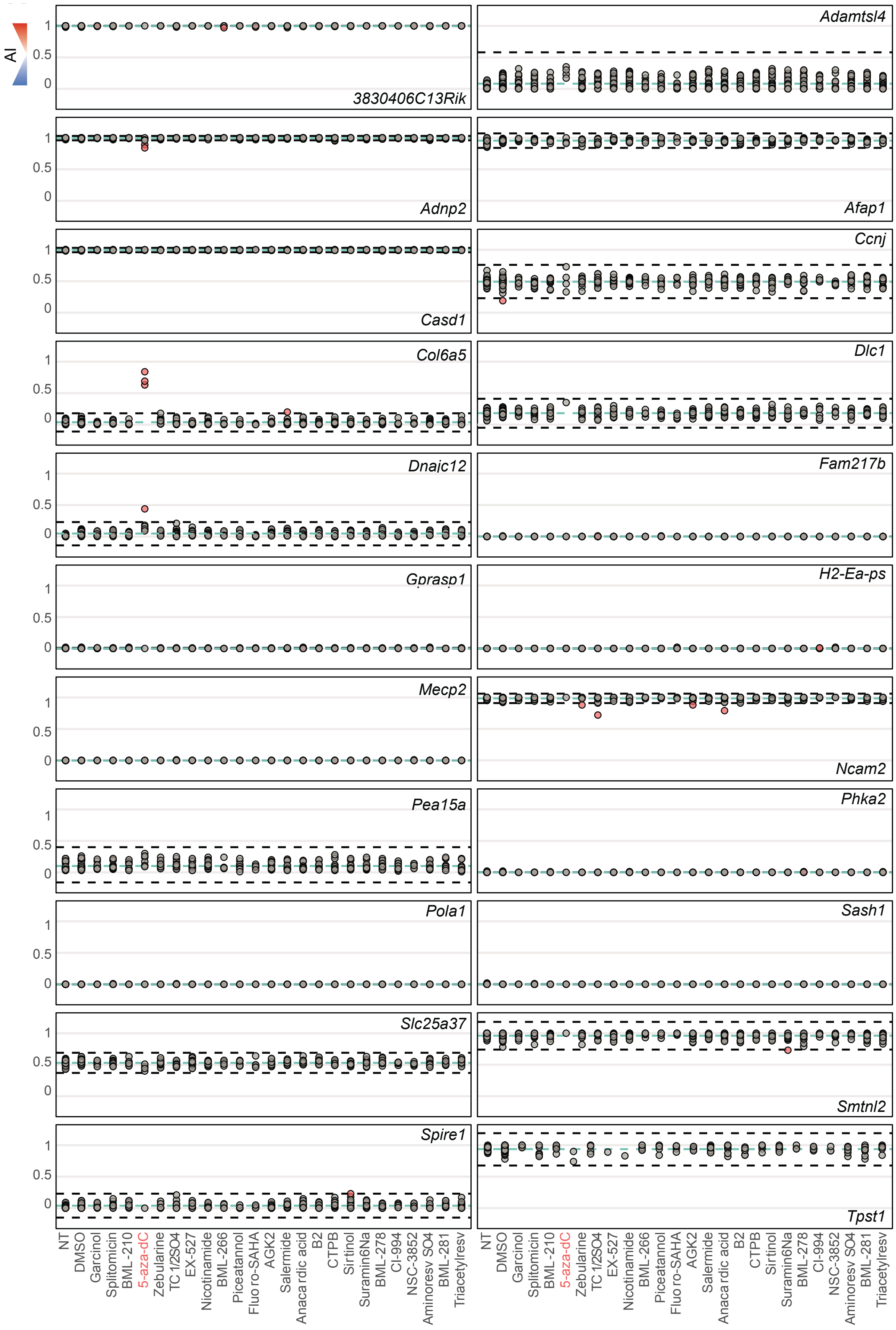

Fig.S3. Screen-seq results for 23 readout genes.
Presentation of complete primary screen results. Each box represents Screen-seq data for a readout gene, with all time-points and concentrations for a single drug shown in the same column. Each point shows AI in one condition. *Blue line*: mean AI for a gene across all conditions; *black dashed lines*: [Q1-3×IQR] and [Q1+3×IQR] (inter-quartile range); *red* points: outlier AI values (hits). See **Suppl. Table S4** for drug hits and **Suppl. Table S5** for complete Screen-seq results. For this analysis, genes with observations with counts under 30 were filtered out (an observation consists of allelic counts for one gene in one well). Since for readout gene *Hdlbp*, we observed counts < 30 for all treatments including control, this gene is not shown in the figure.

Supplementary Figure S4

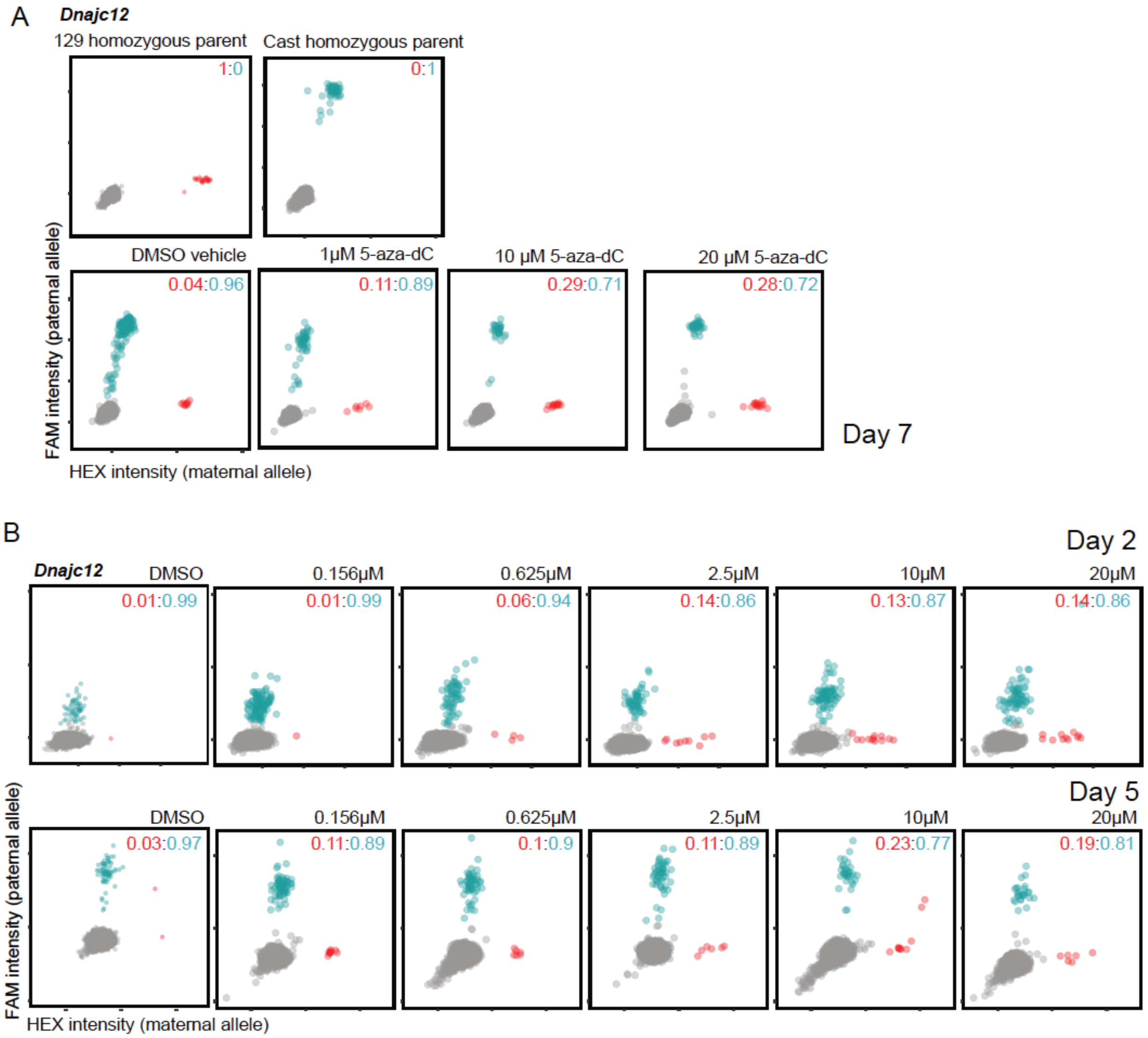

Fig.S4. Relaxation towards biallelic expression in *Dnajc12* allele imbalance in Abl.1 cells on 5-aza-dC treatment.

AI of *Dnajc12* was measured using ddPCR. Scatterplots for 20,000 droplets targeting *Dnajc12* with allele-specific fluorescent probes. in (A) same cDNA samples as used for Screen-seq, and (B) biological replicate experiment in Abl.1 clone are shown. Treatment, DMSO control or 5-aza-dC concentration is shown on top right of the plots. *Grey*: empty droplets; *blue*: droplets with Cast allele amplified (labeled by FAM fluorophore); *red*: droplets with 129 allele amplified (labeled by HEX fluorophore). Ratio of red:blue droplets (AI) is written on the upper right corner within each plot. AI value written in *red* is the maternal AI, shown as a dose-response curve in **Fig. 2B** and **2D**, respectively for A. and B.

Supplementary Figure S5

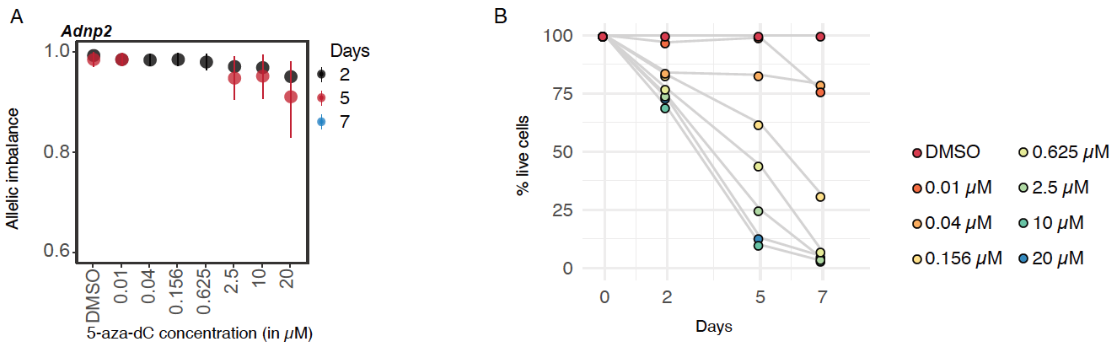

Fig.S5. Dose response curve for Adnp2 and cell viability of Abl.1 clone in biological replicate experiment (A) **Subtle shift in *Adnp2* allele imbalance in Abl.1 cells on 5-aza-dC treatment.** Biological replicate analysis with 5-aza-dC in Abl.1 cells after 2- and 5-days exposure (*black* and *red* points, respectively) using ddPCR. Summary of ddPCR analysis for *Adnp2,* another MAE readout gene that showed up as a hit in Screen-seq on treatment with 5-aza-dC. Concentrations of 5-aza-dC (in μM) are shown on X-axis. (B) **Higher concentrations and longer duration of 5-aza-dC exposure is toxic to monoclonal lymphoid cell line Abl.1.** Percent of live cells (measured by automated Trypan blue assay) was determined for the cells shown in **Fig.2C** and **2D** on the indicated days. Monoclonal lymphoid cell lines such as Abl.1 show ideal growth at of cell densities of 1-2x10^6 cells/ml. For this experiment, these cell densities were not maintained, and overcrowding was observed in wells with DMSO and low concentration of 5-aza-dC, leading to lower viabilities post Day 7. This was modified later for exposure/recovery experiment.

Supplementary Figure S6

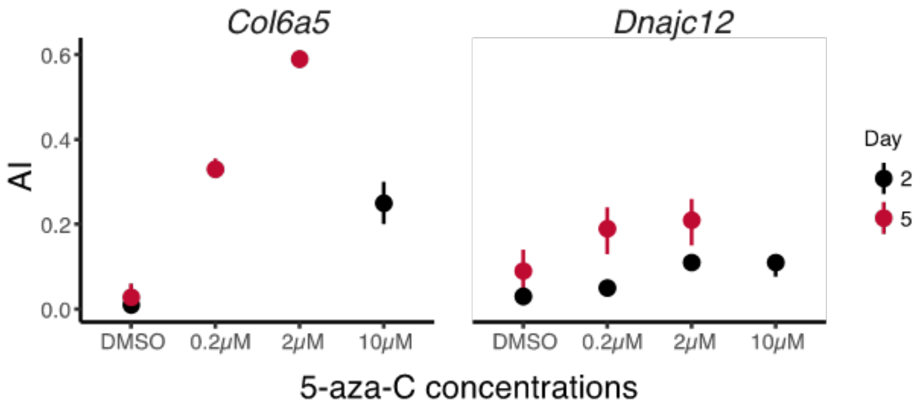

Fig.S6. 5-aza-cytidine (5-aza-C), a closely related compound to 5-aza-dC, causes shifts in allelic imbalance of MAE readout genes *Col6a5* and *Dnajc12* in Abl.1 cells.
Effect of 5-aza-cytidine (5-aza-C), a closely related analog of 5-aza-dC, on AI. AI was measured using ddPCR after treating cells for 2 days (*black circles*) and 5 days (*red circles*) and shown on the Y-axis in Abl.1 cells for *left* – *Col6a5* and for *right* – *Dnajc12* readout genes.

Supplementary Figure S7

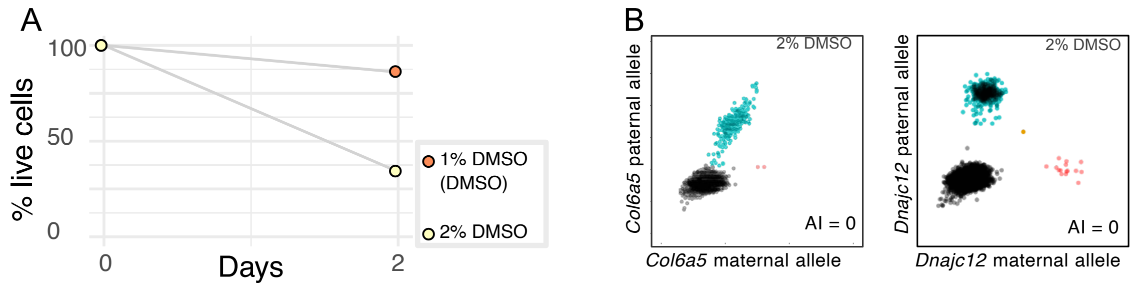

Fig.S7. Inducing cell toxicity in clone Abl.1 by growth in high concentration of DMSO (2%) does not change allelic imbalance of *Col6a5* and *Dnajc12*.

(A) Abl.1 cells were grown in medium with 2% DMSO for 2 days that was equitoxic to high concentrations of 5-aza-dC.

(B) All cells collected on Day 2 were used to measure AI of *Col6a5* (left) and *Dnajc12* (right) using ddPCR. Scatterplots show 20,000 droplets targeting readout genes. *Black*: empty droplets; *blue*: droplets with Cast allele amplified; *red*: droplets with 129 allele amplified. Ratio of red:red+blue droplets shown as AI.

Supplementary Figure S8

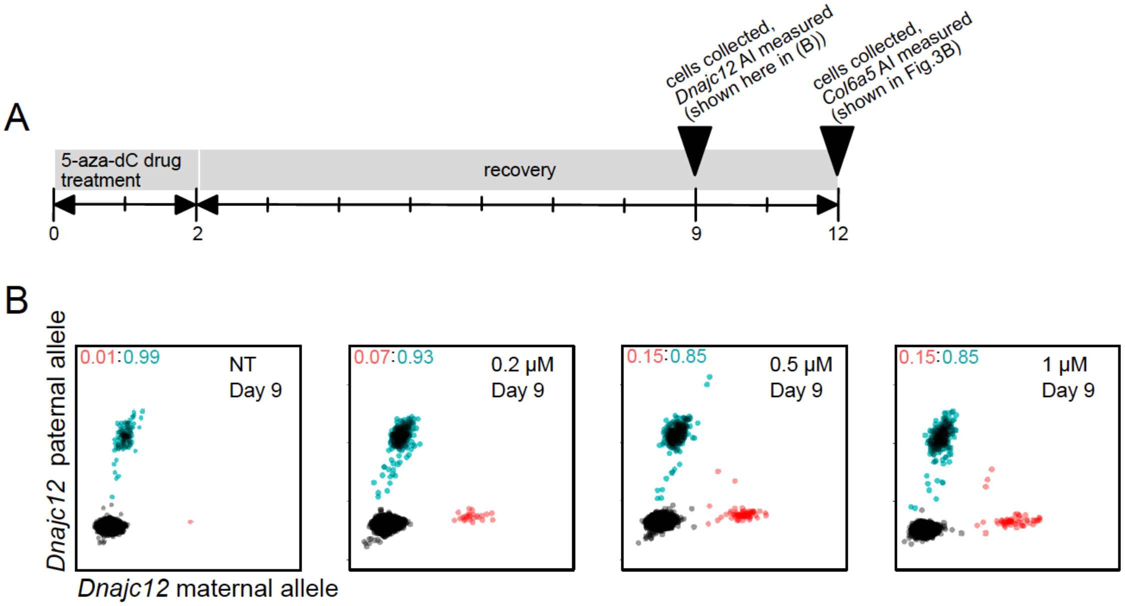

Fig.S8. 5-aza-dC exposure-and-recovery experiment in Abl.1 cells – readout gene *Dnajc12*.

(A) 5-aza-dC exposure/recovery experiment in Abl.1 cells. Cells were exposed to 0.2, 0.5 or 1 µM 5-aza-dC in growth medium for 2 days. Control cells were not treated (NT) with drug and only grown in growth medium. After 2 days, cells exposed to the drug were washed and grown only in growth medium (without drug, called recovery phase) for the rest of the days. Cells were collected on days 2, 5, 7, 9, and 12. For cell numbers on these days measured using automated trypan blue assay, see **Suppl. Table S7.**

(B) AI measurements for *Dnajc12* on day 9 after recovery. Scatterplots for 20,000 droplets targeting the readout gene, *Col6a5*. 5-aza-dC concentration used during first 2 days is shown in the plots. *Black circles*: empty droplets; *blue*: droplets with the Cast paternal allele amplified (labeled by FAM fluorophore); *red*: droplets with the 129 maternal allele amplified (labeled by HEX fluorophore). Ratio of red:blue droplets (AI) is given in the top left of each scatterplot. AI value written in red is the maternal AI.

Supplementary Figure S9

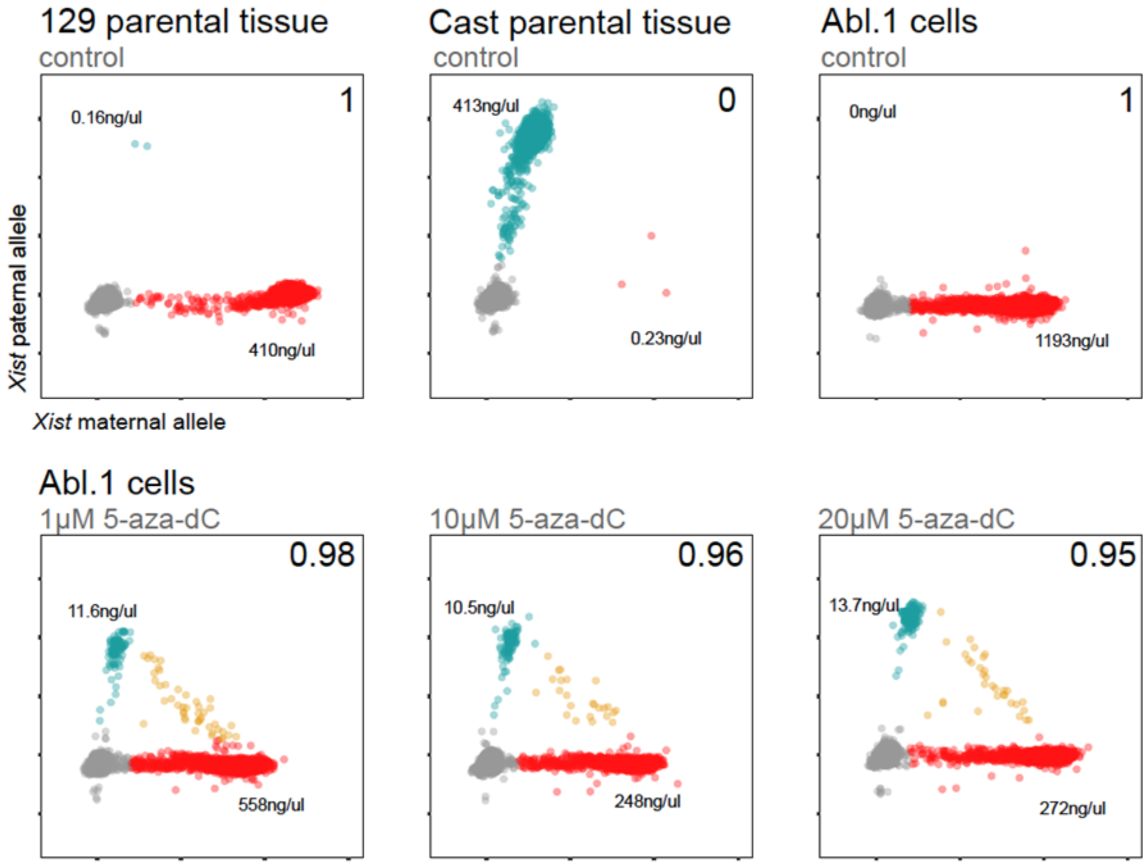

Fig.S9. Change in *Xist* allele imbalance in Abl.1 cells is very subtle on exposure to 5-aza-dC treatment.
AI of *Xist* was measured using ddPCR. Scatterplots for 20,000 droplets targeting *Xist* in control untreated samples (*upper panel*) and 5-aza-dC treated Abl.1 cells (*lower panel*). cDNA samples from day 7 of screening were assessed using ddPCR with allele-specific fluorescent probes. *Grey*: empty droplets; *blue*: droplets with Cast allele amplified; *red*: droplets with 129 allele amplified. Ratio of red:blue droplets (AI) is written on the upper right corner of each plot. Next to red and blue droplets are the copies/µl measured for 129 and Cast *Xist* allele.

Supplementary Figure S10

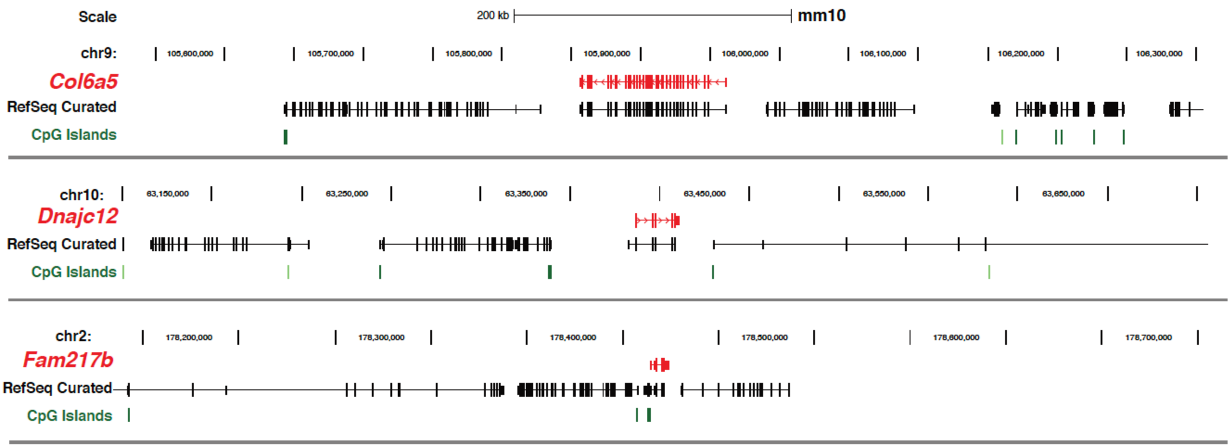

Fig.S10. Changes of allele-specific expression of autosomal genes appear independent of CpG islands.

Comparing the CpG islands in the 200kb genomic region window for readout genes (shown in red) *Col6a5*, *Dnajc12* (genes responsive to DNA demethylation) and *Fam217b* (gene not responsive to DNA demethylation). The CpG island tracks (shown in green) and genomic coordinates were obtained from UCSC browser (mm10).
